# Reasoning in Life: Values and Normativity in Georges Canguilhem

**DOI:** 10.1007/s11196-020-09786-7

**Published:** 2020-11-06

**Authors:** Gabriele Vissio

**Affiliations:** Department of Philosophy and Education Sciences, University of Turin, Turin, Italy

## Abstract

This paper aims at giving an account of the philosophy of norms of Georges Canguilhem in the framework of his philosophical vitalism. According to Canguilhem, vitalism is not a metaphysical or ontological theory, but rather a general attitude or a perspective about life and living beings, both understood employing the axiological concept of ‘normativity’. This notion allows Canguilhem to enlarge the concept of life beyond the field of biological phenomena, encompassing also phenomena of the social world, included technique and scientific knowledge and rationality. Canguilhem’s perspective relocates human activities within a vitalistic conception of life, which redefines the meaning of human reason by putting it in relation to values and norms.

This paper aims at giving an account of the philosophy of norms of Georges Canguilhem in the framework of his philosophical vitalism. Canguilhem develops his normative philosophy within a framework analysis that takes into account the philosophical meaning of the biological and medical concept of ‘norm’. Nevertheless, his work cannot be confined to the narrow field of philosophy of medicine and biology, but aims at providing a wider and more comprehensive perspective, able to provide a philosophical account for more complex aspects of human life, such as knowledge, rationality, and social behaviours.

Even if Canguilhem avoids any reductionist viewpoint, his concepts of vital norms and normativity allow him to interpret human phenomena within a general philosophical approach that has been identified as ‘vitalism’. As it is shown by the first part of this paper (Sects. 1 and 2), this kind of vitalism is quite different from the modern biological theories usually identified by this label, because it relies on a theory of values that provides the basis for his interpretation of life in terms of ‘normativity’ (Sect. 3). This last concept allows Canguilhem to enlarge the concept of life beyond the field of biology and to interpret vitalism as a general philosophy, able to take into account typical human and social phenomena, through a specific idea of rationality that explicitly relates to values and norms (Sect. 4). For this reason, to be a human being means to be a special kind of living being, the one able to reason on values and norms.

## What Kind of Vitalism?

The conceptual label ‘vitalism’ has been used in the modern history of ideas to denote several different scientific, metaphysical, or religious theories and beliefs concerning the status of living beings and life phenomena within the physical unanimated world.^1^ The French philosopher André Lalande wrote, in his *Vocabulaire technique et critique de la philosophie* (1927), that the most general meaning of the word ‘vitalism’ refers to each doctrine or conviction that claims for a radical difference between phenomena concerning life and all other natural (physical or chemical) facts [24]. According to this definition, vitalism often includes (or is associated with) other controversial beliefs, such as psychological and metaphysical theories about the origin of the mind or even animism, intended as a belief in the common origin of life and soul [24].

Since the nineteenth century, a tradition in the history of life sciences pointed out vitalism as an irrational belief and even as an obstacle to the rise and the development of modern scientific biology and medicine. For example, the scientist Emil Du Bois-Reymond (1818–1896) stated, in a speech dedicated to the memory of his former professor Johannes Müller (1801–1858), that vitalism played no positive role in the development of the muscular physiology [20: 135–317]. In so doing, Du Bois-Reymond was failing to take into account the merits of relevant scholars (or at least he was underestimating their work), such as Georg Prochaska (1749–1820), a pioneer of the modern psychological theory of reflexes [12: 139]. Although they were convinced to propose a sort of neutral and positivistic history of science, these scholars forged their historical narrative on an implicit philosophical assumption, identifying the essence of modern scientific reason with a specific philosophical position: mechanism [12: 158]. Furthermore, the negative account of the role of vitalism has been criticized and revised in the first half of the twentieth century by historians and philosophers of science interested in highlighting its positive role in the development of life sciences [11, 12]. Additionally, recent studies are pointing out the complex and multi-layered history of vitalism, trying to bring order in a composed set of different theories, which is difficult to consider as a whole. Within this framework of studies, a very simple but effective taxonomy, proposed by Charles T. Wolfe, identifies three main versions of vitalism [45–47]:

a. A *Substantival* or *Metaphysical Vitalism*, which supposes the existence of a living matter (a substance or a force) that is independent of the physical and chemical laws of inorganic matter.^2^ This kind of vitalism often connects to other metaphysical arguments, such as psychological animism [24], and has strong implications for life sciences. An example of this kind of vitalism is the definition of life as “entelechy” [*Entelechie*] provided by Hans Driesch (1867–1941), especially in his philosophical works *Der Vitalismus als Geschichte und als Lehre* (1905) and then vulgarized in a series of Gifford Lectures in 1907–1908 [18, 19]. The notion of *Entelechie* and Driesch’s vitalism has been the object of a well-known condemnation by some members of the *Wiener Kreis*, such as Moritz Schlick (1882–1936) and Philipp Frank (1884–1966) [45: 356].^3^

b. A *Structural*–*functional Vitalism*, which does not imply the existence of a specific “living matter” or “vital force” and considers living beings as complex phenomena with specific emergent features, different from those regulating inorganic phenomena, claiming a specific viewpoint for the phenomena related to living beings [48, 49]. An eminent example of this vitalism is provided by some representatives of the so-called “*École médicale de Montpellier*”, such as Théofile de Bordeu (1722–1776) or Paul-Joseph Barthez (1734–1806) [48, 49].

c. An *Existential or Attitudinal Vitalism*, which realises that there is a peculiar attitude of a living being towards other living subjects [49]. This specific version of vitalism does not necessarily imply any particular position on the contents of scientific knowledge about life but involves some general consequences at an epistemological level. Examples of this last version of vitalism can be found in the work of the German psychiatrist Kurt Goldstein (1878–1965) [23] and of the French philosopher and historian of science Georges Canguilhem (1904–1995), which is the main subject of this article.^4^

Attitudinal vitalism developed by Georges Canguilhem has been widely analysed by scholars from different perspectives, and they have emphasized its connexions to other sections of Canguilhem’s work: his philosophy of medicine, his work in the history of science [38], his philosophy of techniques [14]. Otherwise, Canguilhem’s vitalism has been also interpreted as a general philosophical programme, a sort of *fil rouge* that allows the interpreter to keep an encompassing outlook on his philosophical production [15, 42]. These two different interpretative approaches are not mutually excluding, but they can reinforce themselves as long as they provide a general interpretive structure that can integrate all of Canguilhem’s philosophical work. Traditionally, several scholars have considered the theory of norms and normativity as an element of continuity in Canguilhem’s work [26, 32, 35], also arguing that the notion of “norm” would play a meta-level role in the theoretical scaffolding of his philosophy [35]. This position appears to match with a valorisation of vitalism since notions of “norm” and “normativity” are core concepts of Canguilhem’s inquiry on health and disease [13].

## Values Beyond Vitalism

Until the mid-1990s, Canguilhem was generally known as an accurate and erudite historian of the modern life sciences—especially biology and medicine—and for his masterpiece in the field of philosophy of medicine, his 1943 thesis *Essai sur quelques problèmes concernant le normal et le pathologique*, lately known, after some integrations in 1966, as *Le normal et le pathologique*. His writings were renowned in a group of experts in these fields, but his influence in the French philosophical culture grew after World War II. In 1994 François Delaporte, a disciple of Michel Foucault (1926–1984) at the Collège de France during the 1970s, edited a collection of essays and texts by Georges Canguilhem with an important Preface written by the American anthropologist Paul Rabinow and a *Critical Bibliography* by Camille Limoges [17, 28]. This bibliography inaugurated a new season for the reception of Canguilhem in the contemporary philosophical culture, and not just because the book was an anthological introduction to his philosophical work. In fact, Limoges entered the list of the several less-known writings Canguilhem wrote during his long career, including an impressive number of texts dating to the 1920s and 1930s. A ‘new’ Canguilhem appeared [3–5], revealing a multifaceted and fascinating landscape of intellectual and cultural references encompassing the *Bildung* of this young philosopher during the 1920s [39, 40] and the emergence of his vitalism between the late 1930s and the beginning of the 1940s [1, 2].^5^

In this multifaceted scene, one of the most prominent figures is represented by the French professor and writer Émile Chartier (1868–1951), mostly known under his penname “Alain”. Chartier played the role of an influential intellectual leader for at least two generations of young thinkers, philosophers, and writers during the first decades of the twentieth century [41]. Professor of philosophy at the prestigious Lycée Henri-IV in the *Quartier Latin* in Paris, this eclectic thinker was a mentor for intellectual personalities like Michel Alexandre (1888–1952) and his wife Jeanne Halbwachs (1890–1980), Simone Weil (1909–1943), Raymond Aron (1905–1983), among the others. In his distinguished book about the “intellectual generation” born around 1905, François Sirinelli dedicated several pages to Alain and his pupils [41, 42], emphasizing the role of Chartier as a “professeur éveilleur”.^6^ Canguilhem was one of the beloved disciples of Alain and he probably was, at least until the mid-1930s, his favourite pupil. He was deeply involved in Chartier’s journal *Libres Propos*, and he took part in different *querelles* defending Alain’s radical pacifism against militarism, but also against different positions in the anti-militarist spectrum.

The political debate after World War I was the framework in which Canguilhem articulated a philosophy of values, which set the basis for his further philosophical inquiries [34]. According to the young Canguilhem, words like ‘war’ and ‘peace’ do not denote facts or factual situations, but a couple of opposing values. In this sense, the Versailles Treaty (1919) claimed to establish peace in Europe as a fact, but under the surface, the war was surviving as a value spreading in society. The compulsory military service, the rhetoric of the winners and the losers, and the general militarization of the civil society [7–9], are just some of the indicators of the permanence of the war in peacetime. The militarist rhetoric depicts the world as a neutral reality, but it is designing society according to specific axiology. According to Canguilhem, even the perception of space and time [6, 10] is socially shaped by the general militarization of society.

More in general, the young Canguilhem was establishing an opposition between the notions of fact and value that would have gained great importance in the forthcoming developments of his philosophy. In particular, between the late 1930s and middle of the 1940s, Canguilhem developed a general philosophy grounded on the notion of value, integrating two other key concepts: norm and life.

## Vital Norms

In his masterpiece *Essai sur quelques problèmes concernant le normal et le pathologique* (1943), Canguilhem proposes to consider life as the ability of the living beings to “judge” elements of their environment or “milieu”, attributing them positive or negative values. In fact, according to Canguilhem:

Even for an amoeba, living means preference and exclusion. A digestive tract, sexual organs, constitute an organism’s behavioural norms. Psychoanalytic language – states Canguilhem – is indeed right to give the name *poles* to the natural orifices of ingestion and excretion. […] There is a dynamic polarity of life [13: 136].

Canguilhem better defines the previous theory of values in the 1943 book, which attributes a ‘polarised’ constitution of reality to values. Furthermore, the notions of subject and subjectivity are now enlarged thanks to the introduction of the concept of the living being, which allows a wider and more general extension of the theory of values beyond the field of the human experience. The Kantian influence of Chartier persists beside a positive reassessment of the philosophy of Henri Bergson^7^—who has previously been the subject of criticism by Canguilhem—and critical reception of some results of the biological and medical philosophical inquiries. In this context, “value” loses its human and moral connotation and becomes a biological concept with a general philosophical meaning. According to Canguilhem, “We can say of the universe of every living thing what Reininger says of the universe of man: […] our image of the world is always a display of values as well” [13: 179].

This general reconsideration of the idea of value and its new arranging in a biological framework goes along with the introduction of the concepts of ‘norm’ and ‘normativity’ at the very heart of the philosophy of Canguilhem. In the 1943 work, Canguilhem states that “*Normative*, in philosophy, means every judgment which evaluates or qualifies a fact in relation to a norm, but this mode of judgment is essentially subordinate to that which establishes norms” [13: 126]. Norms are references for evaluations of factual reality and they constitute criteria for value assignments. Usually, norms are conceived as a matter concerning the human and social world, but in this case “we do not ascribe a human content to vital norms but we do ask ourselves how normativity essential to human consciousness would be explained if it did not in some way exist in embryo in life” [13: 127]. In his essay, Canguilhem takes into account the problem of the meaning of health, disease, and cure, for which he provides normative definitions. There are a social meaning and a specific human way to conceive vital conditions like health, disease, and cure, but these lasts should find their foundation in biological life:

From the sociological point of view, it can be shown that therapeutics was first a religious, magical activity, but this does not negate the fact that therapeutic need is a vital need, which, even in lower living organisms arouses reactions of hedonic value or self-healing or self-restoring behaviours [13: 127].

In this sense, the establishment of vital norms and the consequently polarised evaluation of the surrounding environment (*milieu*) is a typical activity of living beings, well expressed by the pathological condition, which exists only for the living: “Biological pathology exists but there is no physical or chemical or mechanical pathology” [13: 127].

The crucial experience of disease allows Canguilhem to introduce the notion of normativity, one of the most original contributions of his work. If to be alive means to establish norms and evaluate a *milieu*, to be sick is defined as a change in the vital norms of the individual [36]. On this point, the influence of the neurologist and psychiatrist Kurt Goldstein (1878–1965) is explicit and clear: he stated that “disease appears when an organism is changed in such a way that, though in its proper, ‘normal’ milieu, it suffers catastrophic reaction” [13: 185].^8^ “A living being is normal” Canguilhem states “in any given environment insofar as it is the morphological and functional solution found by life as a response to the demands of the environment” [13: 144]. Vital norms are established regimes of life the organism finds by bargaining with his *milieu* and disease is the change of this relationship between the biological individual and the surrounding environment. Nevertheless, a change is not a vanishing: the pathological status is still a living condition, even if an undesirable and unfortunate one, and to be alive means to establish vital norms. In this sense, the sick individual is not devoid of any sort of norms, but it finds itself confined and reduced in its capacity to change these norms. To be sick implies to live in a *milieu* in which I am not able to do certain kinds of things (e.g. I cannot eat a certain food or I cannot do certain physical works, because of a disease or an injury): sickness is a restriction and a limitation of the ability to interact with the surrounding milieu and, precisely because of this, it can be described as a state of restricted ability to negotiate norms with the environment.^9^

Canguilhem calls this ability or capacity to redefine vital norms “normativity” [13: 227]. This concept expresses the active and effective role the living being plays in the relationship with the environment. There is not a unilateral influence of the surrounding environment that mechanically defines the vital norms of the organism, but the organism itself (even the sick organism) can structure the normative relationship with its milieu.

If biological norms exist it is because life, as not only subject to the environment but also as an institution of its environment, thereby posits values not only in the environment but also in the organism itself. This is what we call biological normativity [13: 227].

Even if the term «vitalism» does not appear in the *Essai* for defining his philosophical position, the vitalistic position he would then elaborate from the 1950s relies essentially on the definition of life as normativity and on the idea that living is a form of evaluation.^10^ Especially, in *La connaissance de la vie* (1952) and *La formation du concept de reflèxe aux XVII*^*ème*^ *et XVIII*^*ème*^ *siècles* (1955) and in some other minor writings of that period, Canguilhem began to mature his critical revision of the vitalistic tradition and to develop vitalism as a general philosophical proposal.^11^ This new interest in vitalism did not mean a decrease of interest in the problem of normativity in life and vital norms but on the contrary, a strong integration of this inquiry within a new research framework. The presence of three entire chapters on *Le vivant et son milieu*, *Le normal et le pathologique*, *La monstruosité et le monstrueux* in the third section of *La connaissance de la vie* that opened with one of his most important contribution on vitalism (*Aspects du vitalisme*) is the proof that this two research interests were anything but separated [11].

## Beyond Biology: Human Life Between Social Norms and Rationality

If the definition of life in terms of normativity and polarisation of values allows Canguilhem to ground any human activity as a response to a vital need posed by the relationship between the individual and its milieu, it also requires an enlarged conception of life capable to give an account of vital norms beyond strictly biological explanations.

Nevertheless, Canguilhem seems to be aware of some risks connected to this all-embracing vitalistic position. In a three chapter-long addendum to the 1943 thesis in medicine—the so-called *Nouvelles réflexions sur le normal et le pathologique* (1966)—Canguilhem explicitly takes into account the relationship between social and vital norms, pointing out the risk in those philosophical perspectives that conceive society and social norms based on an organic model [13: 250]. Criticizing social models based on the analogy between society and organic body (e.g. the Comtian theory of social boundary as a “consensus”) Canguilhem argues that social norms are essentially different from biological ones [13: 250]. A biological norm emerges simultaneously to the correspondent biological need, whereas social norms are the result of a confrontation between different social interests and needs [13: 256]. For instance, the urge to feed is necessarily connected to the presence of a digestive system and a nutritional strategy: “in the case of the organism the fact of need expresses the existence of a regulatory apparatus” [13: 252] and this occurs because of a biological need that

has as its center the organism taken in its entirety, even though it manifests itself and is satisfied by means of one apparatus, so its regulation expresses the integration of parts within the whole though it operates by means of one nervous and endocrine system [13: 253].

Conversely, even if society mimics organic life, social needs do not immediately find their solution in a regulatory apparatus that is already part of the same organic body. For this reason, the infringement of a social norm or rule is different from sickness and there is not a social pathology or illness. Actually, “If social norms could be perceived as clearly as organic norms, men would be mad not to conform to them. As men are not mad […] social norms are to be invented and not observed” [13: 259].

Moreover, Canguilhem argues that “the form and functions of the human body are the expression not only of conditions imposed on life by the environment but also of socially adopted modes of living in the environment” [13: 269]. Human beings live in-between two normative orders, which are distinct but not completely independent.^12^ Moreover, there is a single, integrate normative sphere, in which social norms are modes of living in the environment developed by human beings that are also components of the same human–environment. Even if any form of biological reductionism and environmental determinism is excluded, even if social life is conceived as relatively autonomous, social norms respond to a vital need as well.

According to this, in *Aspects du vitalisme*, Canguilhem promoted the idea that every human behaviour, knowledge, and science included, must be considered as an answer given by a special living being to a vital exigency and vitalism itself «translates a permanent exigency of life», which is precisely the necessity to recognise life as the condition of possibility of every kind of human activity [11: 62]. In this sense, even the knowledge of life and its emergence in a material inanimate world, would not have been possible without a living being conceiving that world as an object of interest:

The milieu in which one looks for the emergence of life only acquires its meaning in virtue of the operation of the human living being who takes measurements of it, measurements that bear an essential relation to the technical apparatuses and procedures by which they are made [11: 70].

In this viewpoint, Canguilhem’s vitalism does not aim to explain life as an *imperium in imperio* but rather proposes a monist perspective in which every human relationship with the world can be expressed as an answer to a vital exigency: «If one is to assert the originality of the biological, this must be in terms of the originality of one realm over the whole of experience, and not over islets of experience» [11: 70]. In this sense, Canguilhem’s vitalism is a reaction to those philosophical perspectives arguing for a strict opposition between life and scientific reason. Bergson’s philosophy, for example, claimed for the impossibility of modern science to reach an authentic knowledge of life, because of the analytical and ‘geometrical’ form of scientific intelligence, which he opposes to the philosophical method of intuition; through his philosophical vitalism, Canguilhem aims instead at leading back into unity science and life, retracing in vital normativity the basis of both scientific and social reasoning [35].

## Conclusions

In conclusion, Canguilhem’s vitalism aims at giving an account of typical human activities like knowledge and science grounded in a normative philosophy of life. This allows Canguilhem to fully include human activities and behaviors in a general conception of living phenomena, actually encompassing human life in the ‘natural’ world. This inclusion takes out any temptation to reintroduce an opposition between life and the instrument that makes it possible the knowledge of life itself, namely scientific reason. Through the notion of vital normativity, Canguilhem qualifies life as a domain of values, characterizing human beings as subjects acting in a milieu woven of axiological meanings. In biological life, values are not arbitrarily imposed on the milieu by an absolute subjectivity, but they emerge in a ‘debate’ between the living being and its environment. In a not contradictory, but still different manner, human and social values arise as the product of a special sort of ‘debate’, the one elapsing between distinct parts of the social body. Even though there is no biological obligation, the rational feature of human normativity allows societies to arrange this debate in the form of a confrontation between different exigencies and needs, but also provides to every human subject the possibility to challenge regimes of injustice, claiming for a different order of values.

Therefore, Canguilhem’s philosophical investigation is not confined to the narrow field of philosophy of biology and life sciences, but it could be considered as a general philosophical perspective with important implications for social and cultural sciences. In particular, the description of life as a normative activity could provide sound grounds for the analysis of values and norms as part of the semiotics of culture. To assume this philosophical perspective, for example, could help us to avoid the idea of meaning production as a neutral activity happening in an empty, blank space: on the contrary, semiosphere, intended as the “semiotic space, outside of which semiosis itself cannot exist” [31], should be considered as a tissue of values and axiological polarities. Moreover, according to Lotman, semiospheres are “simultaneously both participant in the dialogue (as part of the semiosphere) and the space of dialogue (the semiosphere as a whole)” [31], a definition that seems to combine well with the Canguilhem’s concept of “milieu”. In this sense, a further and in-depth examination of the semiotic implications of Canguilhem’s thought and of philosophical vitalism is an open line of research, which might provide a sound, original philosophical ground for ensuring the development of semiotic theory and its conceptual tools.

## Data Availability

Not applicable.

## Footnotes

1 For a recent, up-to-date history of vitalism in Modern and Contemporary Age, see Wolfe [45].

2 It is important to note that even though this metaphysical vitalism involves concepts like *anima* or *entelechia* it is not necessarily incompatible with a materialistic point of view. On the contrary, it is possible to trace a history of the emergence of “vital materialism” in modern age [46].

3 The concept of *Entelechie* has been later criticized also because of the controversial ‘political’ meaning Driesch gave to it during the 1930 s [45: 409].

4 About the relationship between Goldstein’s philosophy of the organism and Canguilhem’s vitalism, especially about the issues discussed in this article, see Wolfe [47] and Moya Diez [33]. Another ‘benchmark philosophy’ for Canguilhem’s project of ‘biophilosophy’ is Bergsonism. For some considerations about the relationship between Canguilhem, Henri Bergson (1859–1941), and Gilles Deleuze (1925–1995), who resumed the Bergsonian legacy during the 1960s–1970s, please see [2, 50].

5 For a general presentation of the development of Canguilhem’s philosophy see the work of François Dagognet [16] and the introductive book by Lecourt [25]. A general presentation of the impact of the *Œuvres Complètes* in the current debate on Canguilhem’s philosophy is provided by a recent book by Stuart Elden [21: 10–13].

6 Despite this image of an inspiring mentor, after the World War II and the political experience of Vichy, which involved some of his disciples, Alain and his philosophy were subject of violent controversies, because of the supposed role his radical pacifism played in providing a cultural and political élite prepared to accept collaboration to the Nazi regime [42].

7 There is actually no mention of Bergson in the *Essai*, but we can find a reconsideration of Bergsonism in some text of the same years that are deeply connected to the development of Canguilhem’s normative philosophy of life [1].

8 Quoted by Canguilhem.

9 Nowadays, normativism is one of the fundamental theoretical options in the debate on definitions of “health”, “sickness”, “illness” and “disease” in philosophy. About the position of Canguilhem’s definitions of “norm” and “normativity”, the main bibliographical reference is the book by Élodie Giroux [22]. On this topic see also the articles by Lingua [30] and Pilotto [37].

10 In this sense, some scholars have explicitly identified Canguilhem’s concept of illness with a vitalistic perspective [36] and underlined its connection to his theory of values [44].

11 Camille Limoges has drawn attention to the project of a *philosophie biologique* Canguilhem developed starting from the 1950s [29] and Charles Wolfe and Andy Wong have interpreted Canguilhem’s project of a biophilosophy as a «return to vitalism» [50]. On this point see also the article by Vissio [43].

12 According to this perspective, some scholars have tried to propose a philosophical anthropology based on Canguilhem’s notion of norm and normativity. An eminent example of this kind of attempts are the works by Guillaume Le Blanc [27].
